# Spring-assisted posterior vault expansion: a parametric study to improve the intracranial volume increase prediction

**DOI:** 10.1038/s41598-023-48143-z

**Published:** 2023-12-04

**Authors:** Lara Deliège, Karan Ramdat Misier, Dulanka Silva, Gregory James, Juling Ong, David Dunaway, Noor Ul Owase Jeelani, Silvia Schievano, Alessandro Borghi

**Affiliations:** 1 University College London, London, UK; 2 Great Ormond Street Hospital, London, UK; 3 Department of Engineering, Durham University, DH1 3LE Durham, UK

**Keywords:** Biomedical engineering, Computational models

## Abstract

Spring-assisted posterior vault expansion has been adopted at the London Great Ormond Street Hospital for Children to treat raised intracranial pressure in patients affected by syndromic craniosynostosis, a congenital calvarial anomaly causing the premature fusion of skull sutures. This procedure involves elastic distractors used to dynamically reshape the skull and increase the intracranial volume (ICV). In this study, we developed and validated a patient-specific model able to predict the ICV increase and carried out a parametric study to investigate the effect of surgical parameters on that final volume. Pre- and post-operative computed tomography data relative to 18 patients were processed to extract simplified patient-specific skull shape, replicate surgical cuts, and simulate spring expansion. A parametric study was performed to quantify each parameter’s impact on the surgical outcome: for each patient, the osteotomy location was varied in a pre-defined range; local sensitivity of the predicted ICV to each parameter was analysed and compared. Results showed that the finite element model performed well in terms of post-operative ICV prediction and allowed for parametric optimization of surgical cuts. The study indicates how to optimize the ICV increase according to the type of procedure and provides indication on the most robust surgical strategy.

## Introduction

Craniosynostosis (CS) is a birth defect characterized by the complete or partial premature fusion of one or more cranial sutures. This condition affects 1 in 2000–2500 live births worldwide and exists in two forms: syndromic and non-syndromic^1^. The latter is the most common condition encountered in paediatric craniofacial surgery^2^, involving a single suture, usually either the sagittal, the coronal (“unicoronal” when one suture is involved, “bicoronal” when both are involved), metopic or lambdoid suture. Syndromic craniosynostosis (SC) accounts for 15% of the total cases^3^ and occurs in conjunction with other anomalies that typically include underlying genetic mutations^4^ and therefore can be grouped in specific phenotypes. The syndromic cases are classified into different syndromes: Apert and Crouzon are the most commonly encountered. Apert syndrome is characterized by bicoronal synostosis causing temporal widening and occipital flattening, as well as symmetrical syndactyly of the hands and feet^5^. The Crouzon syndrome generally includes brachycephaly due to the bicoronal synostosis, and shallow orbits resulting in exorbitism^4^. Other types of SC like multi-sutural craniosynostosis exist but are not linked to a specific genetic diagnosis. The degree of head deformity in SC patients varies, but overall, these patients require complex care.

Craniosynostosis is treated surgically: the age of the patient, the number and location of fused sutures can determine the surgical management strategy^6^. The need for this treatment is both for cosmetic and functional reasons. Indeed, many SC patients experience raised intracranial pressure (ICP) with serious consequences on vision and brain development^7^. The main aim of the surgery is to improve the final head shape for aesthetic reasons but also to increase the volume of the cranial vault to allow a healthy development in more severe cases. Spring assisted posterior vault expansion (SAPVE) is a procedure adopted in Great Ormond Street Hospital (GOSH) for reshaping and enlarging the calvarium of patients affected by SC. The surgery involves a skin incision over the top of the head from ear to ear, exposing the calvarium. An osteotomy is performed just behind the coronal sutures in order to free the posterior portion of the skull. Springs (elastic distractors) are inserted and fixed using notches, created on both side of the osteotomy, and the skin is then closed on top. The springs allow to dynamically reshape the skull and release pressure over time due to the viscoelastic nature of the paediatric calvarium^8^. The GOSH springs are prefabricated torsional springs with an initial dimension (opening) of 60 mm between the tips and they exist in three models (S10, S12, S14) having increasing stiffness (0.17, 0.39, 0.68 N/mm, respectively) depending on the wire diameter (1.0, 1.2, 1.4 mm, respectively)^9^. The SAPVE surgical outcome is uncertain due to the complexity of the procedure and the lack of knowledge of the interaction between springs and skull. Finite Element (FE) modelling proved suitable to accurately predict the outcomes of spring expansion in syndromic and non-syndromic populations^8,10,11^. A previous work developed, tested, and validated a three-dimensional FE simulation platform to predict the ICV increase for SC patients, using full anatomical reconstructions retrieved from pre- and postoperative computed tomography (CT) scans. Results were assessed visually and numerically using postoperative images and ICV measurements^11^.

The long term ICV increase observed ranges between 10 and 20%^12,13^: the ideal ICV increase is still debated, and it is unclear how surgical parameters affect cranial augmentation. So far, the number and location of the springs as well as the osteotomy position is performed according to the operating surgeon's judgement: indication on the optimal surgical strategy would prove extremely valuable to operating surgeons in preoperative planning of SC patients’ treatment.

At GOSH, an ICV augmentation of 20% is considered ideal as a smaller increase would lead to a sub-optimal outcome meaning the procedure was unsuccessful and the patient will need a second cranial vault expansion surgery.

In this work, we have developed a parametrizable FE model based on a previously validated study to assess the impact of the surgical parameters (osteotomy’s dimension and springs location) on the predicted surgical outcome in terms of ICV increase. The results will provide a better understanding of the effect of surgical strategy adoption and will ensure optimal outcome will be reached, avoiding the need for revision surgeries at a later stage.

## Methods

### Patient population, image processing

Eighteen patients (age at surgery = 2.6 ± 1.8 years) who underwent SAPVE at GOSH were retrospectively recruited for this study. All patients had received preoperative (44 ± 60 days before surgery) and postoperative (147 ± 144 days after surgery) CT scans. The population was divided into three groups according to the SAPVE surgical strategy: one group where 2 springs were implanted on top of the skull (group T2, n = 6), one group where 2 springs were implanted laterally (group L2, n = 6) on the skull and one final group where 4 springs were implanted, two on top, two laterally (group TL4, n = 6). Each patient received 2 or 4 springs of the same model (either S10, S12 or S14^9^). Osteotomy location and dimensions, spring models and positions were extracted from postoperative CT scan reports and operative notes^11^. Similarly, to our group’s previous work^11^, DICOM images were imported into Simpleware ScanIP (Synopsys, Mountain View, CA) to separate the calvarium from other tissues using a grayscale threshold method ([230,3020] HU—pre-defined in Scan IP for bone segmentation). All the 3D reconstructions obtained were scaled to account for head growth between the day of the preoperative imaging and the day of surgery using ICV growth curves published by our group^14^. This study was approved by the Ethics Committee of the Great Ormond Street Hospital for Children (Ethical approval number: UK REC 15/LO/0386). All research was performed in accordance with relevant guidelines and regulations. Informed consent was obtained for all imaging data from all patients and/or their legal guardians. This study protocol was approved by the Great Ormond Street Hospital and Great Ormond Street Institute of Child Health joint Research and Development Office (internal R&D number 20DS33). All procedures performed in this study were in accordance with the 1964 Helsinki declaration and its later amendments or comparable ethical standards.

### Models (surface, anatomical) creation

For each patient, a first model (A—anatomical-model) was created by extracting the anatomy directly from the preoperative CT (following the same methodology as Deliège et al.^11^). The use of the CT-based full anatomical models for the SAPVE outcomes prediction was validated in Deliège et al.^11^. A second model (S—surface-model) was created by simplifying the anatomical model: the outer layer of the anatomical model was extracted and smoothed in Meshmixer (Autodesk Inc., San Rafael, CA); all holes and artefacts, apart from the foramen magnum, were processed to obtain an even shell. Such model was then carefully aligned to have the horizontal plane passing through standardized anatomical landmarks (the cranial nasion and the left and right auditory meatuses). Each aligned model was then imported in Solidworks 2018 (Dassault Systems, France) and converted into a NURBS (non-uniform-rational-b-spline) surface (“surface model”).

**Figure 1:**
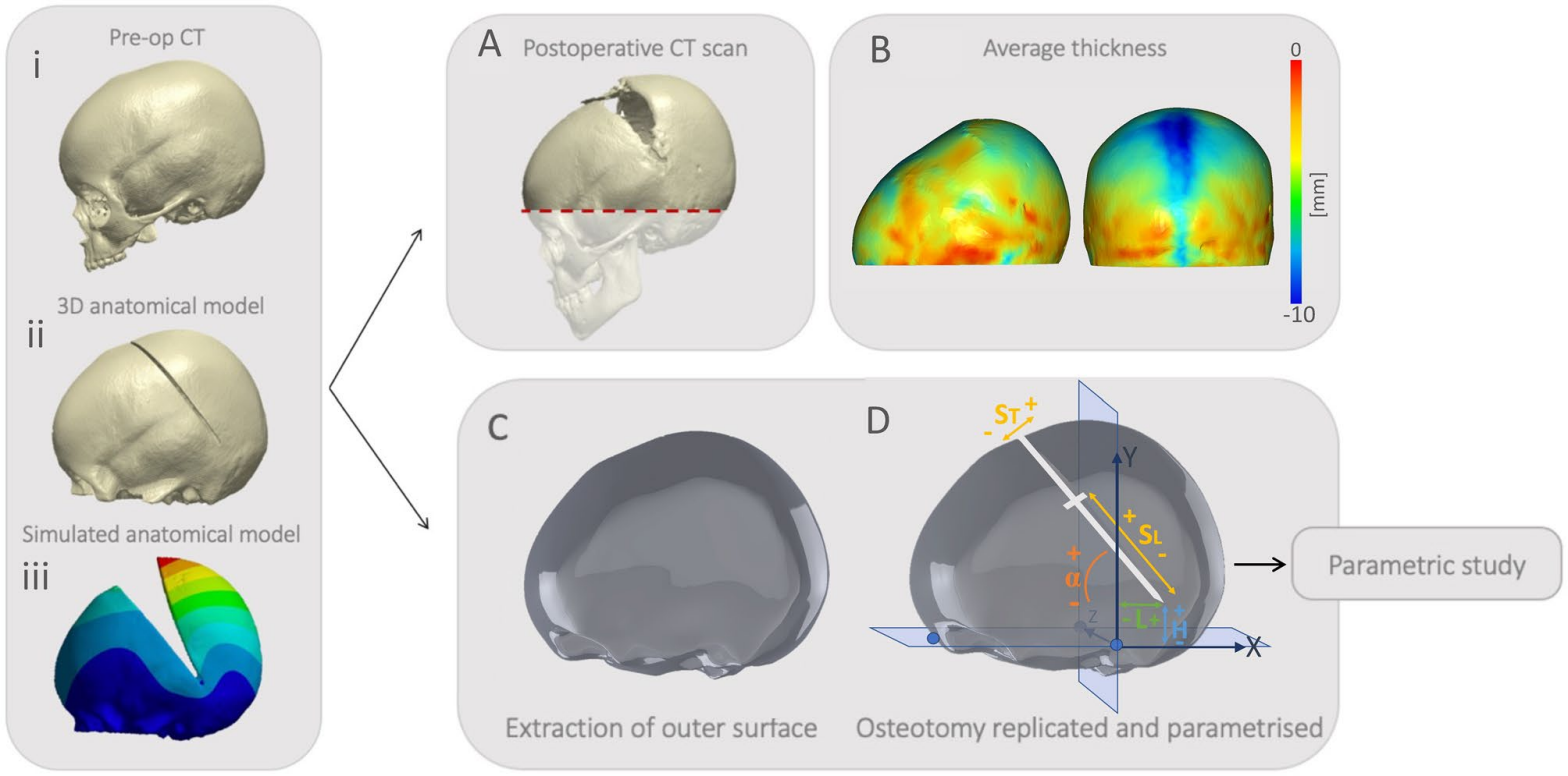
(From left to right) Framework illustrating the modelling framework: pre- (**i**), post-operative, (**ii**) CT images from SAPVE patient and (**iii**) 3D simulation. (**A**, **B**) Calculation of skull thickness using postoperative imagery. (**C**) Extraction of external surface of preoperative CT scan and (**D**) parametrization of osteotomy leading to the parametric study and sensitivity analysis.

The osteotomies were replicated based on the postoperative images and pre-recorded operative notes. These surgical cut locations were identified using a reference frame common to every models. The origin was set in the left auditory meatus, at the intersection of two planes; the horizontal plane passing through the nasion and both auditory meatuses and the vertical plane, perpendicular to the first plane and through the auditori meatuses. Those axes define our reference frame as the x and y axes lie on the horizontal and vertical plane respectively (Fig. 1D). The z axis is defined as the line at the intersection of both planes to guarantee a symmetrical cut in our model.

For each patient, the osteotomy cut was created along the x–y plane. The bottom end of the osteotomy was described using parameter L (the anterior–posterior position on the horizontal axis—positive towards the back of the skull) and H (the cranio-caudal position on the vertical axis—positive towards the cranial part of the skull); *α* was the angle between the osteotomy and the horizontal plane (positive in the clockwise direction) like illustrated in the framework (Fig. 1). The location of the lateral springs’ notches was parametrized using another parameter (S_L_)—the distance between the spring notches and the bottom end of the osteotomy—positive towards the front of the skull). For procedures involving springs placed on the top of the skull (T2 and TL4), the springs positions were described using parameter S_T_ the distance between the notches and the midline plane (positive towards the right side of the skull).

### Simulation of spring deployment

Simulation of spring expansion for the A model was carried out similarly to Deliège et al., 2021^11^. For the S models, each model was imported into Ansys mechanical 2020 R1 (Canonsburg, Pennsylvania, US). Each model was manually assigned with a specific thickness assumed constant throughout the calvarium. This value was retrieved by calculating the average thickness between the skull’s inner and outer surfaces of the relative postoperative CT scans using Simpleware Scan IP. Each geometry was discretized using linear tetrahedral elements. The optimal mesh order was fixed upon the previous mesh dependency analysis^11^. However, a new independence test was done on a subset of the population as the original model was simplified and mesh density could be decreased (originally averaging ~ 500.000 elements, reduced to ~ 60.000 element). The foramen magnum’s outline was constrained in every direction to avoid rigid motion^15^.

For both models, the stainless-steel springs were modeled using node-to-node linear spring conditions implemented in ANSYS, applied on opposite notches as reported in^8,9,16^. The calvarium bone of all patients was modeled as an isotropic elastic material with Young’s Modulus E = 1300 MPa (based on the average age of 2 years for our population^17^, Poisson's ratio *ν* = 0.22^18^). As in a previous work from our group^8,10^, a viscoelastic model was adopted to mimic the skull reshaping over time, due to the springs’ action. Optimized relaxation properties found in previous work were used^11^.

### ICV extraction

The ICV value for the A model was manually extracted similarly to Deliège et al. The ICV value for the S model was automatically computed by an approximation tool scripted in IronPython using the Ansys Customization tool. This script calls an external function able to calculate the volume of the 3D convex hull that contains the model’s mesh.

### Parametric analysis

A response surface analysis was performed for the whole population to assess the impact of the surgical parameters (*L*, *H*, *α, S*_*T*_ and/or *S*_*L*_) on the simulated post-expansion ICV. A Design of experiment approach (DoE—an engineering technique to minimize the number of experiments necessary to characterize parametric sensitivity of a mechanical system^19,20^ was used by varying each parameter within a pre-defined patient-specific interval: the ANSYS optimal space-filling DoE method was adopted to produce a number of parameter combinations independent from the number of parameters and provide uniformly spread samples on the pre-defined parameter intervals (Ansys 20.2 user manual). The variation bounds were defined as a percentage of the overall anatomical dimensions i.e., skull height, length, and width. For each patient, the intervals for L and H were fixed at [− 10%; + 10%] of the total skull length and height, respectively. The parameter α which represents the inclination angle was assigned a fixed interval of [− 20%; + 20%] based on the initial surgical configuration. This interval was adopted as it encompasses as many possibilities as possible while staying in a realistic practical range. S_T_ and S_L_ were allowed a 3-cm movement and the two springs placed on top of the skull were only permitted to move symmetrically i.e., simultaneously move closer together or further apart. For each patient, either 25 or 27 simulations were performed depending on the number of springs implanted (2 and 4 springs, respectively) for a total of 462 simulations for the whole population.

A local sensitivity (which accounts for the sensitivity of the output variable—the ICV—to the variation of each input variable) chart was extracted for each patient. The software calculates the change of outputs based on the change of inputs independently, when all the other parameters are set to a specific value (Ansys 20.2 user manual).

### Statistics

Each parameter’s sensitivity values were averaged for the whole population to compare the results depending on the procedure performed and assess which parameters have the most influence on the final ICV gain. Statistical differences were assessed using the Wilcoxon-rank test (*p* < 0.05 was assumed significant).

## Results

### FE model validation: post-operative shape and ICV

**Figure 2:**
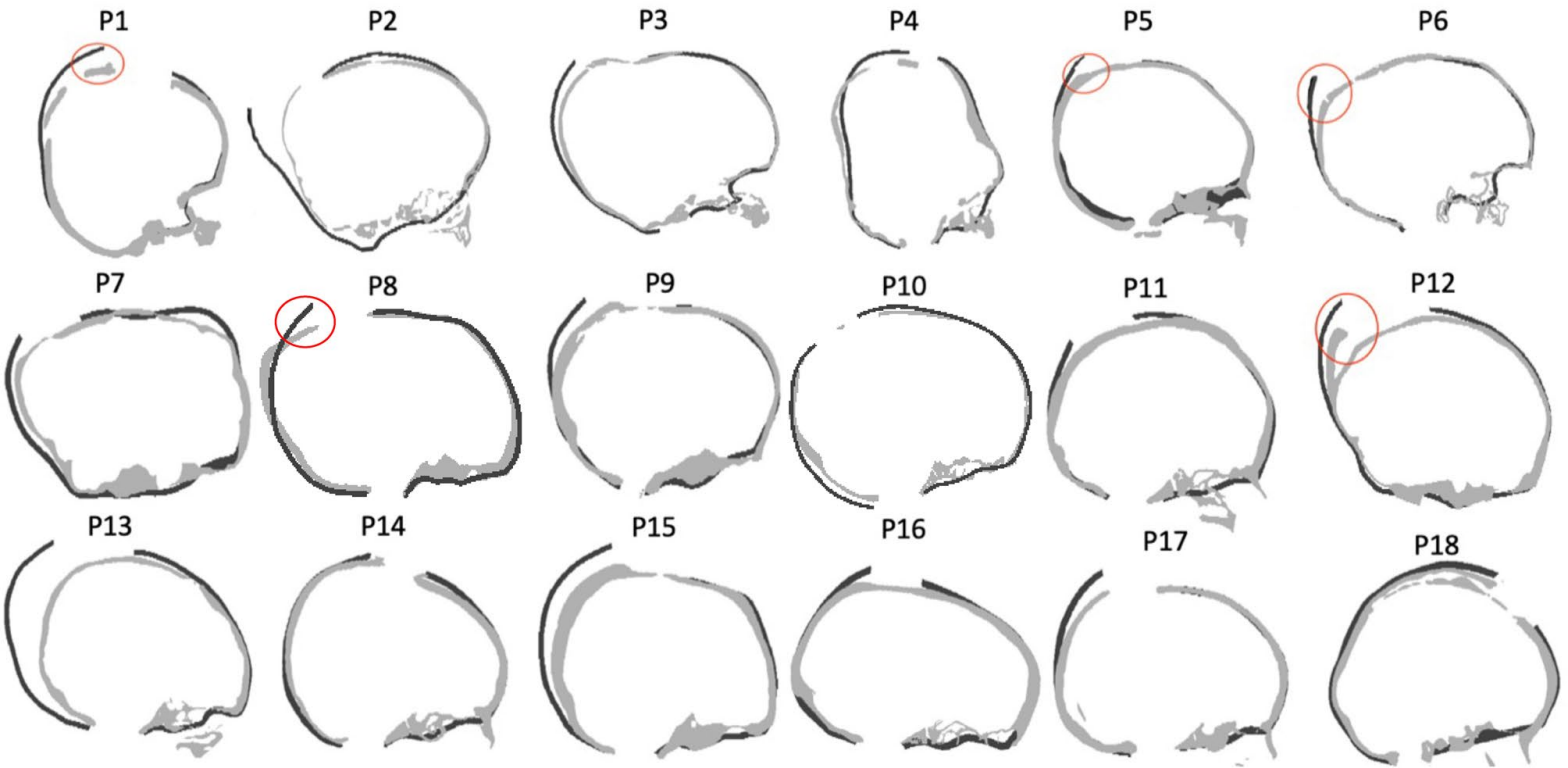
Illustrations of the comparison between the simulated models (dark grey) and the post- operative CT scan (light grey). Discrepancies due to the on-table remodeling is highlighted in red.

**Figure 3:**
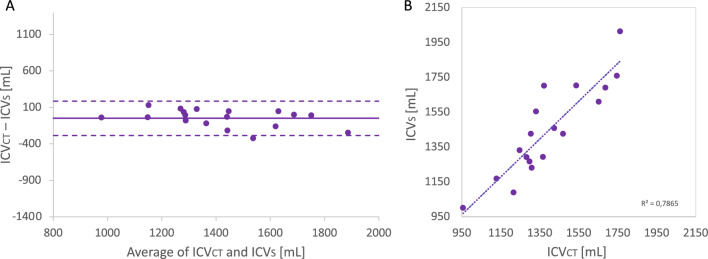
(**A**) Bland–Altman plot showing a comparison between post-operative ICV measured from 3D scans and that predicted ICV from FE simulations for each patient. (**B**) Correlation between the two measurements.

For all 18 patients, simulations for both A and S models were run with the initial osteotomy placement and spring positions extracted from postoperative images to validate the new methodology. Figure 2 shows the sagittal and transverse cross-sections of the simulated end-of-expansion calvaria (dark grey) of the S model, in comparison with the postoperative skull shapes extracted from postoperative CT scans (light grey): in this case, the FE model reproduced the postoperative skull shape closely in most cases, whereas for a subset of patients (1, 5, 6, 8, 12), the model showed high deviation on the top of the parietal bone (highlighted with circles in Fig. 2). When the ICV of the S model population (ICV_S_) was compared with the postoperative ICV extracted from postoperative CT scans (ICV_CT_), an average difference of − 49.96 ± 120.17 ml was found: The average postoperative ICV recorded from CT scans was 1394 ml ± 210 ml and the FE model yielded comparable values with an average of 1444 ml ± 257 ml (r = 0.89, p < 2 × 10^–6^, Fig. 3). ICV values for the FE model retrieved automatically (via python code), were validated by comparing the results with the relative postoperative measurements manually calculated in Meshmixer (R^2^ = 0.99). The ICV_S_ was also compared with the ICV of the A model population (ICV_A_) and a good correlation (r =  + 0.91, p = 0.0001) was found between ICV_A_ and ICV_S_. When comparing ICV_A_ and ICV_S_ with ICV_CT_, a slightly higher error margin was found (7% ± 6%) for the S model compared to the A model (6% ± 4%).

### Parameters’ sensitivity

The impact of the change in surgical parameters (osteotomy—α, H, L—and springs position S_T_ and S_L_) on the post-expansion ICV was assessed.

**Figure 4:**
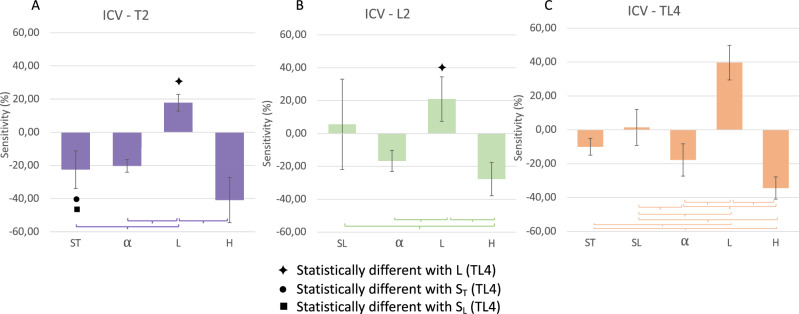
Local sensitivity (%) of the ICV to each surgical parameter, for 3 different groups: (**A**) 2 springs on top (T2), (**B**) 2 springs on the side (L2), (**C**) 4 spring inserted (TL4).

Figure 4 displays the average local sensitivity of the post-expansion ICV to each parameter for the 3 groups: the results show similarities throughout the cohort but highlight some differences depending on the type of procedure adopted. For all groups, a negative sensitivity to the osteotomy angle (the smaller the angle, the larger the postop ICV), a positive sensitivity to the L parameter (the more posterior the osteotomy, the larger the postop ICV), a negative sensitivity to the H parameter (the more caudal the osteotomy, the larger the postop ICV) were found. Negative and positive average sensitivity to S_L_ (3.64%) and S_T_ (− 16.28%) were found. Figure 4 summarizes the ICV sensitivities for each group to the surgical parameters.

**Figure 5:**
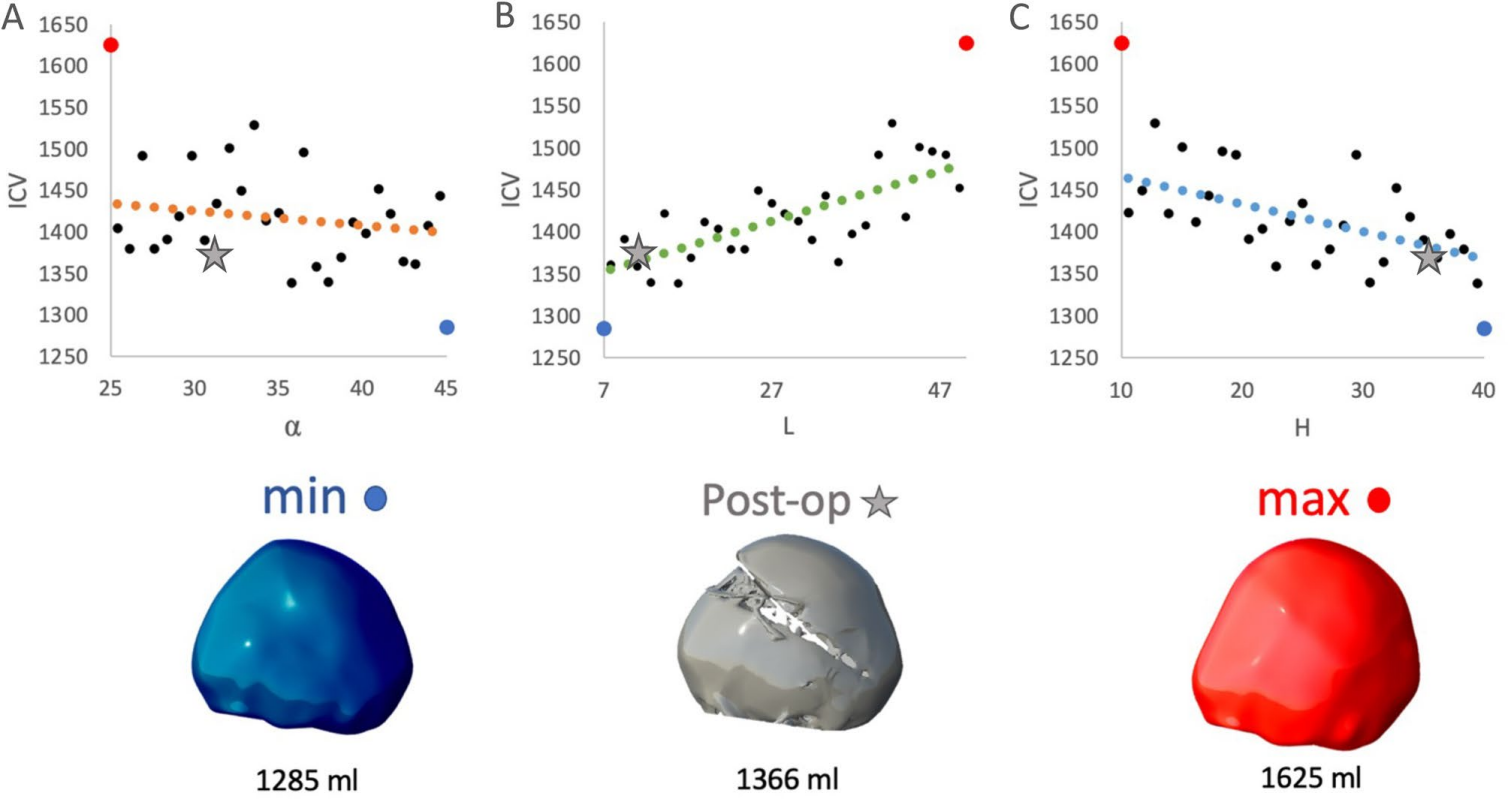
Representative patient: variation of post-operative ICV for the 3 main parameters: (**A**) α, (**B**) L, (**C**) H. Minimal (blue dots) and maximal (red dots) volumes achievable in the DoE for the parametric ranges. Grey star reports the ICV achieved post-surgery obtained with the osteotomy configuration adopted by the surgical team on the day of spring insertion.

For a representative patient, Fig. 5 displays the variation in postoperative ICV for the 3 main parameters (*α*, *L*, *H*). The blue and red dots represent the minimal and maximal volumes (respectively) achievable in the DoE for the parametric ranges defined, while the grey star reports the ICV achieved post-surgery in relationship to the osteotomy configuration adopted by the surgical team on the day of spring insertion.

The α and H parameters showed very similar behavior for the three groups. When four springs were used (group TL4), the results showed that the anterior–posterior position (parameter *L*) of the osteotomy had a higher effect in terms of postoperative volume maximisation (39.65 ± 10.20) compared to the T2 (17.82 ± 5.02, p = 0.01) and L2 group (14.98 ± 10.31, p = 0.03).The top springs *S*_*T*_ location shows a more pronounced sensitivity for the T2 group (− 22.53 ± 11.35 compared to the TL4 group (− 10.03 ± 4.94, p = 0.03). The L2 group had a wide variation in ICV sensitivity to the lateral springs S_L_ location (range 41.28 to − 38.13), while the TL4 had a very low sensitivity to it (range 13.11 to − 13.43).

## Discussion

The SAPVE procedure not only focuses on correcting abnormal head shape in patients affected by SC, but it also helps normalising intracranial volume^21^ and improve neurological development^22^. Due to those implications and their unpredictable outcomes that could lead to a risk of needing a revision surgery, the preoperative planning for this surgery proves to fundamental to ensure the best treatment outcome.

The DoE carried out in this study allowed the creation of many different surgical configurations and, along with a response surface analysis, granted a better understanding of the surgical parameters’ effect on the final volume. We were able to highlight which surgical parameters would have the largest influence on the cranium expansion according to the procedure performed (i.e., SAPVE with 2 springs on top of the skull, 2 springs on the side or 4 springs implanted).

The sensitivity analysis highlighted a similar behaviour for the 3 populations with overall an increase of the ICV with higher values of the parameter L, lower values of parameters α, H and caudal spring location. Although comparable trends appeared throughout the cohort, a standout local sensitivity was noted for two parameters from different groups: the ICV is significantly more sensitive to the parameter L in the TL4 group as well as to the S_T_ parameter for the T2 group.

Very few clinical papers report precise details on osteotomy position in SAPVE procedures: De Jong et al.^23^ indicates that, for SAPVEs, the most caudal horizontal part of an L-shaped osteotomy is positioned just above the transverse sinus and torcula for safety reasons. An osteotomy too close or below the torcula may increase the risk of transverse venous sinus injury. However, Zapatero et al.^24^. compared the risks of venous bleeding between a low or high posterior cranial osteotomy in the case of posterior vault distraction osteogenesis. The conclusion indicated that, although infratorcular osteotomy was associated with greater blood loss and transfusion per kilogram of body weight, it didn’t show a greater risk of injury and allowed a bigger ICV gain compared to a high osteotomy. Our results validate this statement as the ICV is inversely proportional to the osteotomy height; the lower the surgical cut, the bigger ICV gain^24^.

While this study shows promising results, some of the limitations stated in previous works are still currently applicable until further investigation of the condition-specific properties: the same young’s modulus (1300 MPa) was used throughout the population despite the age ranging from 8 months to 5.5 years. The value adopted was established on the average age of 2 years. It is however known that the calvarial stiffness increases with age therefore the skull stiffness of the youngest patients of this cohort is likely to be inferior to that value. Furthermore, the literature data on cranial stiffness used for the FE modelling is relative to healthy subjects; the mechanical properties of patients affected by syndromic craniosynostosis are—to date—unknown.

Additionally, this work included a relatively small population, due to the limited number of patients who had both pre- and postoperative CT scans available at GOSH. A recent study from our group^25^, showed that the ICV in syndromic patients correlates with the head surface volume. Therefore, in perspective works, non-ionizing imaging methods such as 3D surface scanning could be used to perform both preoperative planning and postoperative assessment and overcome the need for CT data in the modelling pipeline. Although the prediction error between the predicted FE and the postoperative models reconstructed from CT images remains within a rather small range (8 ± 7%), larger discrepancies are still noted for several patients. It is useful to note that, for a subset of patients (patients 1, 5, 6, 8, 12), the superior part of the occipital bone was remodelled by the surgeons on the operation table using plates and screws. This cannot be predicted a priori by the simulated model, resulting in a slightly over-estimated ICV. However, this modification could be manually included a posteriori to the modelling so the ICV increase can be further amended.

Finally, the average thicknesses retrieved from the post-operative CT images were proven less accurate for three patients (2, 13, 15), for whom the average thickness taken over the entire skull is smaller than the actual thickness of the occipital bone (i.e., less resistance to the springs force), leading to a larger expansion. This will be taken into account in further studies where the localised thickness changes due to possible bony ridges and skull base thickening will be considered^26^. It is currently a limitation of the use of shell elements which does not allow the definition of a location dependant thickness. Future works will also investigate the use of a syndromic population curve to estimate the appropriate thickness from the patients’ age.

In this study, our aim is to explore potential optimization of surgical parameters, while we did not investigate different procedures, unlike Cross et al., who did explore such alternatives for the correction of sagittal synostosis on the same patient. However, this methodology could eventually be applicable to other spring-assisted procedures; preliminary work on the computational modelling of vertical vector PVE procedure^27^ is on-going, and already showing promising results in terms of ICV and skull shape prediction. This type of procedure has been recently adopted at GOSH for similar patient cohorts; future publications will investigate the results of both models to highlight not only the most impactful surgical parameters but also the best procedure in terms of functional and aesthetic outcomes.

## Conclusion

The results analysis of this study highlighted the optimal osteotomy position to maximize the volume gain and reduce the risk of needing a second corrective surgery. These outcomes showed a larger expansion when the osteotomy is positioned further back and as low as safely possible for any procedure. When only 2 springs are implanted on top of the skull, the position of those springs significantly more impactful and greater ICV gain is obtained when the distance between them is increased. Finally, the analysis suggests that procedures with two springs implanted on the side of the skull should be avoided as this cohort showed the least consistency and the more spread. This may indicate that the outcomes of this surgery are more sensitive to the parameters and therefore less predictable. Overall, procedures involving 2 springs (on top or side of the skull) show more sensitivity which suggests that the insertion of 4 springs would a more robust choice of surgery.

## Data Availability

The data that support the findings of this study are available on request from the corresponding author, LD. The data are not publicly available due to patient privacy.
